# Evaluation of Volatile Profile and *In Vitro* Antioxidant Activity of Fermented Green Tea Infusion With *Pleurotus sajor-caju* (Oyster Mushroom)

**DOI:** 10.3389/fnut.2022.865991

**Published:** 2022-04-14

**Authors:** Wei-Ying Su, Shu-Yi Gao, Si-Jia Zhan, Qi Wu, Gui-Mei Chen, Jin-Zhi Han, Xu-Cong Lv, Ping-Fan Rao, Li Ni

**Affiliations:** ^1^Institute of Food Science and Technology, College of Biological Science and Engineering, Fuzhou University, Fuzhou, China; ^2^Food Nutrition and Health Research Center, School of Advanced Manufacturing, Fuzhou University, Fuzhou, China

**Keywords:** antioxidant activity, green tea, *Pleurotus sajor-caju*, submerged fermentation, volatile components

## Abstract

Green tea has distinct astringency, bitter taste, and typical green flavor because of its post-harvest treatment without withering and enzymatic oxidation. Microbial fermentation has been identified as a promising strategy that could give green tea infusion a special taste flavor. This might be linked to the metabolic transformation ability of microorganisms. In this study, starter culture of edible mushroom *Pleurotus sajor-caju* (oyster mushroom) was used for submerged fermentation of green tea infusion in order to improve its flavor and taste quality. The volatile profile determined by headspace solid-phase microextraction, coupled with gas chromatography mass spectrometry, showed that the contents of (*Z*)-2-penten-1-ol and methyl heptadienone in green tea infusion were decreased significantly by the fermentation with the basidiomycete *P. sajor-caju* (*p* < 0.01), which would alleviate the herbal and grass flavor of green tea infusion to a certain extent. Meanwhile, the contents of linalool and geraniol were increased 9.3 and 11.3 times, respectively, whereas methyl salicylate was newly produced after fermentation by *P. sajor-caju*, endowing the fermented tea infusion with a pleasant flower and fruit aroma. In addition, the polyphenol profile was determined using high-performance liquid chromatography equipped with ion trap mass spectrometry, and the results indicated that the contents of most polyphenols in green tea infusion decreased significantly after fermentation by *P. sajor-caju*. The reduction of catechins and anthocyanins in fermented green tea infusion alleviated the astringency and bitterness. Moreover, the antioxidant activity of fermented green tea infusion was obviously decreased, especially the DPPH-free radical-scavenging ability and the ferric-reducing power. However, it is noteworthy that the ABTS-free radical scavenging ability was improved compared with the unfermented one, indicating that the increased tea pigments and volatile metabolites (such as linalool and geraniol) after fermentation with *P. sajor-caju* may also contribute to the antioxidant capacity of fermented green tea infusion. Overall, the innovative approach driven by *P. sajor*-caju fermentation has achieved promising potential to manipulate the green tea flavor.

## Introduction

Green tea is very popular in most of Asia, especially in China and Japan, because it contains more bioactive compounds (e.g., polyphenols) than black tea, which makes it have even greater benefits (1). However, green tea usually has a distinct astringency and bitter taste, as well as a typical green flavor, mainly due to its post-harvest treatment without withering and enzymatic oxidation (2). A variety of catechins, especially epigallocatechin gallate (EGCG) and epigallocatechin (EGC), play a key role in the distinct astringency and bitterness of green tea drinks. It is urgent to carry out deep processing to develop more diversified products and expand the product's category and sales market of the green tea. Fermented foods are known for their good nutritional and health benefits. Microbial fermentation is a promising and effective strategy to improve low-grade green tea, and it not only gives green tea infusion a special taste and flavor, but also produces certain physiological effects to promote health. It has been reported so far that the microorganisms used for the fermentation of tea infusion mainly include *Lactobacillus* and *Acetobacter* in bacteria (3–5), yeasts and molds in fungi (6–9), and edible fungi (10, 11).

Edible fungi belong to the classification Eumycophyta. Most of them are Basidiomycotina, whereas a few are Ascomycotina (12). Basidiomycetes are considered to be a promising tool because they can synthesize natural flavor compounds and rich extracellular enzymes, and can be used to regulate the sensory properties of beverages and foods in the food industry. A lot of research evidence has shown that basidiomycetes can be used to develop alcoholic drinks and plant beverages. It was previously reported that wine, beer, and sake were fermented by using the characteristics of ethanol dehydrogenase produced by *Tricholoma matsutake* and *Pleurotus ostreatus*, and the fermented products have the physiological functions of preventing cancer and thrombosis (13). The two techniques of *Grifola frondosa*-fermented soybean milk and *Ganoderma lucidum*-fermented pumpkin juice can obviously improve the bad flavor of plant beverages produced by thermal processing. According to the inspiration of the above two techniques, it was found that basidiomycete fermentation can improve the flavor and taste of tea. In a previous study, fresh tea was used as a substrate to obtain a new type of fermented tea with a good flavor through solid-state fermentation of *G. frondosa* and *G. lucidum* (14). *Flammulina velutipes* was reported to produce the characteristic flavor compound 2-ethyl-3,5-dimethyl pyrazine, creating a nutty and chocolate-like floral odor impression after low-grade *Longjing* green tea fermentation (11). It was also reported that *Wolfiporia cocos* (Fu Ling) fermentation changed the characteristic green grass smell of green tea infusion into a strong fragrance of flower and jasmine flower, and significantly improved the flavor quality of green tea (10). Moreover, beverages fermented by basidiomycetes can have some functional activities, such as antibacterial, immune regulation, and antioxidant (15, 16). At the same time, the rich extracellular enzyme system of basidiomycetes will produce some unique flavors in beverage production (15, 17, 18).

As a typical basidiomycetes, *Pleurotus sajor-caju* (oyster mushroom) has high nutritional values, therapeutic properties, and a variety of environmental and biotechnological applications (19). The fruiting body and mycelium of *P. sajor-caju* are rich in vitamins and amino acids and other nutrients. Besides, it has been widely used as a traditional medicine because of its various bioactive properties, including antitumor, antioxidant, and antimicrobial properties (20, 21). It especially possesses a distinctive flavor, in which a series of aliphatic components such as 1-octen-3-ol and 3-octanone are the main flavor active components contributing to the “fungal flower” and “fruity taste” (22). Phenolic compounds are among several compounds that have been proved to have antioxidant effects on the scavenging-free radicals present in the body (23, 24). The most abundant phenolic compounds reported in edible mushrooms belong to the phenolic acid family. In addition, *P. sajor-caju* can use various by-products of the food industry as a growth matrix to secrete a variety of extracellular enzymes, including phenol oxidase, peroxidase, and glucosidase, which are closely related to the formation of flavor quality (25–29). However, the effects of the fermentation with *P. sajor-caju* on the volatile flavor composition and antioxidant activity of green tea infusion have not been investigated.

Headspace solid-phase microextraction technology (HS-SPME) was used to extract the volatile components in fermented tea infusion, and gas chromatography with mass spectrometry (GC-MS) was used to measure and compare the difference between volatile components in green tea infusion before and after fermentation with *P. sajor-caju*. In this study, the high-performance liquid chromatography with mass spectrometry (HPLC-MS) was used to compare the differences in polyphenols caused by fermentation processing. ABTS- and DPPH-free radical-scavenging abilities and the ferric-reducing antioxidant power were used to evaluate the *in vitro* antioxidant activity of green tea beverage produced by the fermentation with *P. sajor-caju*.

The aim of this study was to investigate the effects of *P. sajor-caju* fermentation on volatile profile and *in vitro* antioxidant activity, and to provide feasible technical support and innovative approaches for the deep processing of low-grade green tea. The results of the research play an important guiding role in the development of new green tea beverages in China and even in the world.

## Materials and Methods

### Chemicals and Materials

*P. sajor-caju* CGMCC 5.593 strain was purchased from the China General Microbiological Culture Collection Center (CGMCC) and deposited at the Fuzhou University Institute of Food Science and Technology. Green tea (Fuyun No. 6) was purchased from the market. Potatoes were purchased from the local supermarkets. Agar powder, anhydrous glucose, soy peptone, yeast extract powder, vitamin B1, KH_2_PO_4_, MgSO_4_, and NaCl were obtained from the Sinopharm Chemical Reagent Co., Ltd. 2-Octanol was purchased from the German Dr. Ehrensorfer company. All were analytically pure.

### Preparation of Green Tea Infusion

Tea and boiling water were prepared according to the material ratio of 1:30 and leached in a water bath at 95°C for 15 min. The filtrate was divided into 50 ml/250 ml (filtrate volume/conical flask volume), pasteurized at 80°C for 30 min. The tea infusion was stored at −20°C until usage.

### Culture of Strain

Liquid seed medium for *P. sajor-caju* CGMCC 5.593 contains 3% glucose (*w*/*v*), 4‰ yeast extract powder (*w*/*v*), 4‰ soy peptone (*w*/*v*), 1‰ KH_2_PO_4_ (*w*/*v*), 0.5‰ MgSO_4_ (*w*/*v*), and 0.05‰ vitamin B1 (*w*/*v*). Distilled water was used as a solvent, boiled, divided into 50 ml/100 ml (filtrate volume/conical flask volume), and sterilized at 121°C for 20 min (27). Inoculation shovel was used to take a fungus block of approximately 0.5 × 0.5 cm^2^ from the original strain *P. sajor-caju* CGMCC 5.593, which was maintained on potato dextroseagar (PDA) slants to the plate with about 20 ml PDA medium. The plate was cultured in a constant temperature incubator at 28°C until the mycelium covered the whole plate and was stored at 4°C. A hole punch with a diameter of 14 mm was used to take a block of mycelium from the plate and then added block to the liquid seed medium of *P. sajor-caju*. The medium was incubated on a rotary shaker (28°C, 200 rpm) for 6 days in darkness to get a sufficient amount of mycelium.

### Fermentation of Tea Infusion

The fermentation process parameters of tea infusion referred to the methods described by Zhang et al. (12). In detail, *P. sajor-caju* mycelia were prepared from 30 ml pre-cultured seed fermentation broth and collected by centrifugation (4,000 rpm, 2,150 × *g*, 10 min, 20°C) and washed twice with sterile water. The collected *P. sajor-caju* mycelia were resuspended in sterilized tea infusion to obtain a stock culture at a concentration of 0.3 g/ml. The stock culture was then inoculated into an Erlenmeyer flask (250 ml) containing 50 ml tea infusion at a 10% inoculation volume. The fermentation was carried out at 28°C for 3 days under aerobic conditions on a rotary shaker (200 rpm) in the dark (28). During the fermentation, the detection solution of the samples obtained by means of centrifugation (4,000 rpm, 2,150 × *g*, 10 min, 4°C) was used for GC-MS analysis, sensory evaluation, and antioxidant activity determination.

### Sensory Evaluation

The sensory evaluation method referred to previously has been described with minor modifications (30). Six odor qualities (herbal, green, floral, fruity, sweetish, and toasty) and three taste qualities (bitter, sweet, and astringent) were selected to describe the flavor of the tea infusion before and after fermentation. The unfermented and fermented tea infusions were preheated at 80°C and packed 15 ml in teacups (capacity, 20 ml; bottom diameter, 32 mm; top diameter, 45 mm). The detection solution was evaluated by the sensory panel (5 men and 7 women). They were invited to rate the given odor qualities of the unfermented and fermented tea infusion according to a five-level intensity scale, which ranged from 1 to 5 (1 = very weak, 2 = weak, 3 = medium intense, 4 = strong, and 5 = very intense). Finally, the sensory score was averaged and analyzed by *t*-test.

### Headspace Solid-Phase Microextraction

The HS-SPME for the extraction of volatiles from the fermented tea infusion is a valuable technique that can effectively trap volatile compounds in various samples present on the surface of the synthetic organic fiber polymers (31, 32). For HS-SPME, divinylbenzene/carboxen/polydimethylsiloxane fiber (50/30 μm, 2 cm, SUPE- LCO, USA) in combination with a manual solid-phase microextraction injection handle were used. The fiber was aged by the gas chromatography inlet at 230°C for 30 min before use. Then, 2 g NaCl, 6 ml detection solution, and 10 μl 2-octanol at a concentration of 1 mg/l (as an internal standard) were added into a 15-ml screw-top headspace vial (33). The samples were preheated at 50°C for 10 min, followed by headspace extraction, at the same temperature for 30 min. Finally, the analytes were directly desorbed in GC-MS inlet at 230°C for 15 min.

### Gas Chromatography Mass Spectrometer

Gas chromatography was carried out using an Agilent 7890-B chromatograph equipped with an Agilent 5977A quadrupole mass spectrometer. The column used was a polar 30 m × 0.25 mm i.d., 0.25 μm (HP-INVOWAX, USA). Helium (99.999%) was used at a constant flow rate of 1 ml/min as the carrier gas. According to the method of Wang et al. (34), the chromatographic heating procedure was as follows: the initial temperature was 40°C, held for 4 min; then, the temperature was raised to 230°C at 5°C/min and held for 3 min. The mass spectrometry conditions were as follows: electron ionization energy, 70 eV; ion source temperature, 230°C; quadrupole temperature, 150°C; scan mode, TIC; and scan range, *m*/*z* 33–550. The volatile peaks were identified by matching the National Institute of Standards and Technology 11 mass spectral database and the retention index (RI, determined by n-alkanes C7–C40). As it is difficult to collect all the standards of the identified volatile compounds, a semi-quantification with a single internal standard of 2-octanol was used in this study, as described by Liu et al. (35).

### LC-MS Analysis

The samples in this experiment were taken from the fermented tea sample on the zeroth and third day of the blank group, as well as the tea infusion on the third day of fermentation. The sample was detected after passing through the 0.22 μm filter membrane. Referring to the methods described by Ana López-Cobo et al. (36), the HPLC system consisted of a C18 column (4.6 × 250 mm, 5 μm; Waters, Milford, MA). The mobile phase, consisting of 0.1% FA (v/v) in water (A) and 100% ACN (B), was used at a flow rate of 1 ml/min. The injection volume was 10 μl. B gradient program was set as follows: 0–4 min, from 10 to 15%; 4–5 min was linear gradient increased to 16%; 5–8 min, linear gradient increased to 18%; 8–12 min, linear gradient increased to 20%; 12–13 min, linear gradient increased to 22%; 13–14 min, increased to 25%; 14–16 min, gradient increased to 28%; 16–17 min, increased to 30%; 17–18 min, linear gradient increased to 31%; 17–18 min, linear gradient increased to 31%; 18–19 min, linear gradient increased to 32%; 19–24 min, linear gradient increased to 50%; 24–27 min, linear gradient increased to 75%; 27–36 min, linear gradient decreased to 10%; and 36–38 min, isocratic at 10%. Column temperature was set at 25°C, and the detection wavelength was 254 nm. The mass spectrometer was operated in negative modes with an electrospray ionization spray voltage of 4 kV, gas temperature of 210°C, gas flow of 8l/min, and full-scan range of 50–1,100 (*m*/*z*).

### Determination of Antioxidant Activity

The antioxidant activity was carried out by three assays, namely, 2,2′-azino-bis-3-ethylbenzthiazoline-6-sulphonic acid (ABTS) free radical-scavenging rate, 2,2-diphenyl-1-picrylhydrazyl (DPPH) free radical-scavenging rate, and ferric iron-reducing antioxidant power (FRAP). ABTS-free radical-scavenging activity (7 mM in H_2_O) contains 2.45 mM potassium persulfate. The tea sample (about 200 μl) was mixed with 800 μl ABTS-free radical solution for 6 min at 30°C, and the absorbance was determined at 734 nm. DPPH is a stable free radical molecule of red color. About 100 μl tea samples were mixed with 100 μl of DPPH (0.2 mM) in methanol, and after 30 min incubation at room temperature, the absorbance at 517 nm was read. The FRAP assay was performed with 100 μl tea sample, then 500 μl phosphate buffer (0.2 M, pH 6.6) and 500 μl 1% K_3_Fe(CN)_6_ was added and place in a water bath at 50°C for 20 min. After cooling, 500 μl of 10% trichloroacetic acid was added and allowed to stand at room temperature for 10 min. Then, 500 μl of the above reaction solution was taken, 500 μl deionized water and 100 μl 0.1% ferric chloride solution were added, and the absorbance at 517 nm was read. The detailed approaches of antioxidant activities have been described by Li et al. (37).

### Statistical Analysis

The experimental data were expressed as a mean of three separate determinations using Excel. The significant changes between the groups were assessed by SPSS software (version 22.0, 1989, IBM, USA) with *t*-test and one-way ANOVA. Heatmap analysis, hierarchical clustering analysis (HCA), and principal component analysis (PCA) were done using R software (version 3.6.1).

## Results

### Sensory Evaluation of Tea Infusion Before and After Fermentation

**Figure 1:**
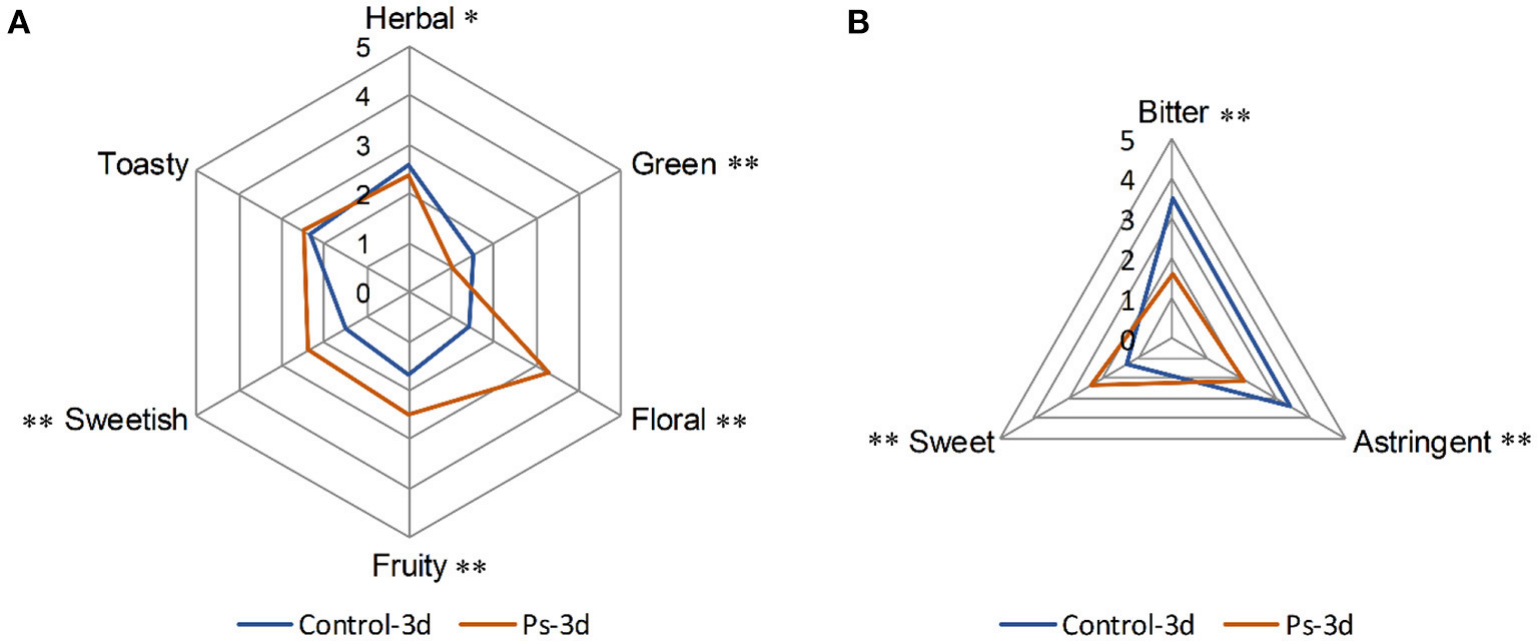
Comparative flavor profile analysis of the aroma **(A)** and taste **(B)** in green tea infusion before and after fermentation by *Pleurotus sajor-caju* (Control-3d: unfermented green tea infusion; Ps-3d: green tea infusion fermentation for 3 days; *p value of before vs. after fermentation was lower than 0.05, **p value of before vs. after fermentation was lower than 0.01).

To compare the aroma and taste changes of tea infusion by fermentation of *P. sajor-caju*, a panel of 12 assessors evaluated the scent of each sample in the descriptors herbal, green, floral, fruity, sweetish, and toasty. Meanwhile, three flavor attributes, including bitter, sweet, and astringent, were evaluated in each sample. Till 3-day fermentation, the aroma and taste in green tea infusion varied in both its quality and intensity (Figure 1). The fermented tea infusion changed in a favorable direction that the floral, fruity, and sweetish scents significantly increased (*p* < 0.01) while the odors of herbal (p <0.05) and green (*p* < 0.01) significantly decreased (Figure 1A). Additionally, fermented green tea displayed a significantly lower intensity (*p* < 0.05) of bitter and astringent attributes (Figure 1B).

### Changes of Volatile Components in Tea Infusion After Fermentation

**Figure 2:**
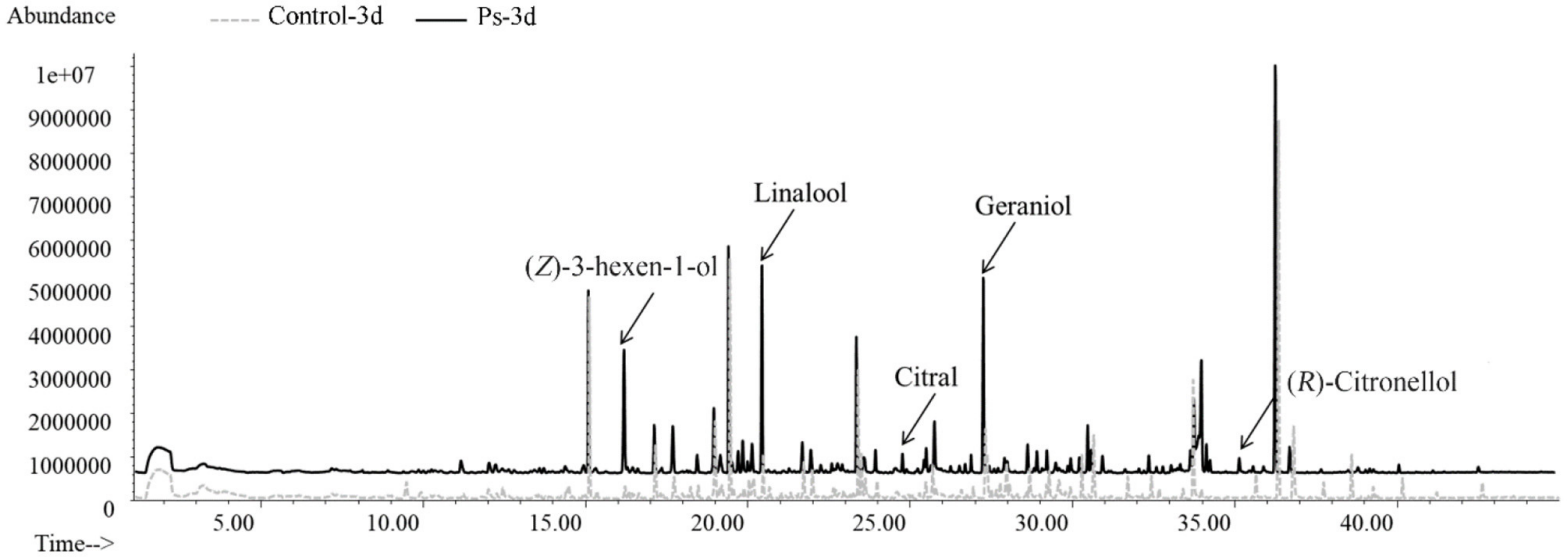
Gas chromatography total ion chromatogram of volatile components in tea infusion before and after fermentation.

**Figure 3:**
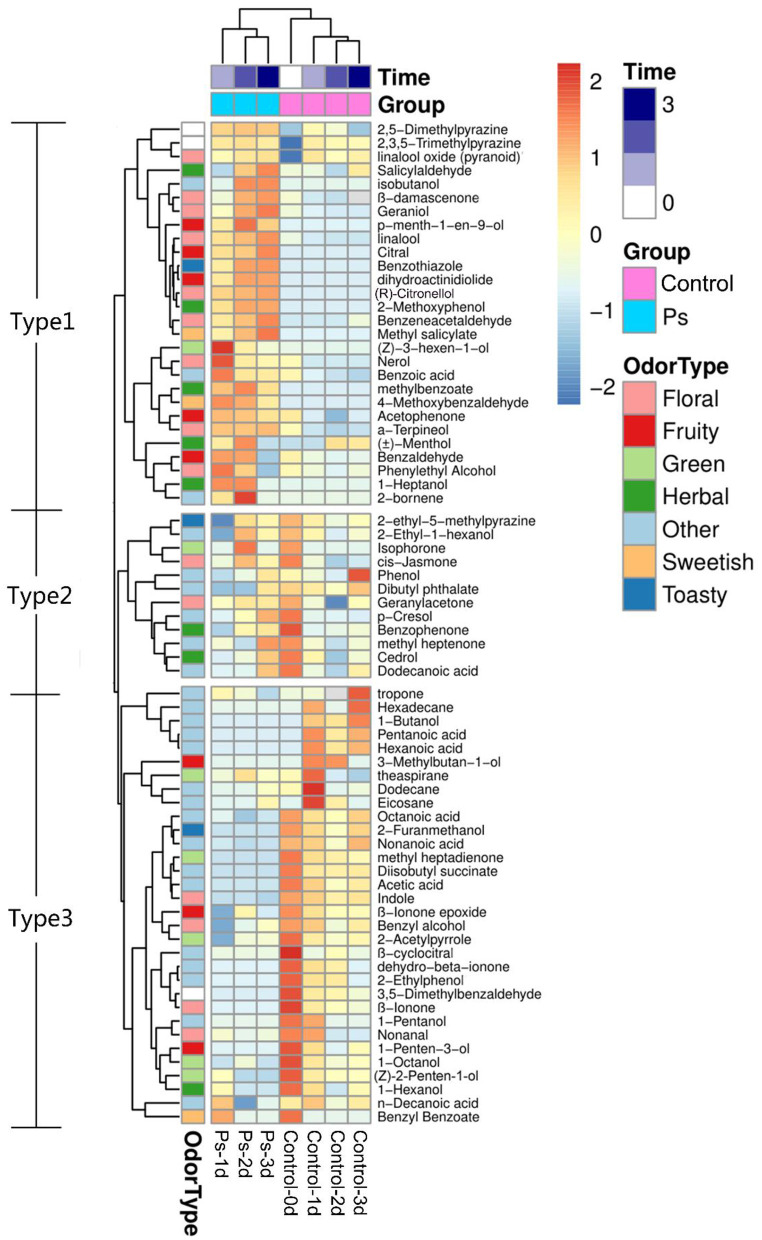
Heatmap of volatile components of green tea infusion before and after fermentation (Control: unfermented green tea infusion; Ps: green tea infusion fermented by *Pleurotus sajor-caju;* time: from 1 to 3 days).

By applying HS-SPME/GC-MS, a total of 72 substances were identified in green tea infusion before and after fermentation, including 5 hydrocarbons, 23 alcohols, 8 aldehydes, 12 ketones, 6 esters, 8 acids, 6 benzenes, 1 pyrrole, and 3 pyrazines. As shown in the total ion flow diagram obtained by gas chromatography (Figure 2), the fermentation made little difference to the overall peak. However, there was a significant increase in the content of substances fermented by *P. sajor-caju*, mainly including (*Z*)-3-hexen-1-ol, linalool, citral, geraniol, and (*R*)-citronellol. These substances present floral and fruity fragrance, which are beneficial to enhance the flavor. Moreover, the heatmap analysis revealed that there was a remarkable difference between the unfermented and fermented groups for presenting two characterized clusters correspondingly (Figure 3). These volatile components can be further divided into three groups, namely, significantly higher experimental group (type 1), no significant difference (type 2), and significantly higher control group (type 3). Type 1 contained 28 substances, of which 12 chemicals were added, such as methylbenzoate, citral, (*R*)-citronellol, methyl salicylate, nerol, and dihydroactini-diolide. In addition, the amount of (*Z*)-3-hexen-1-ol, benzaldehyde, α-terpineol, linalool, geraniol, β-damascenone, and phenethyl alcohol increased greatly. Type 2 consisted of 12 substances, such as geranylacetone, *cis*-jasmone, and phenol. Type 3 comprised 32 substances with 12 chemicals disappearing after fermentation, including β-ionone, (*Z*)-2-penten-1-ol, methyl-heptadienone, hexanoic acid, 2-furanmethanol, and 1-hexanol. Furthermore, the contents of benzyl alcohol, 1-octanol, theapirane, indole, and other substances were significantly reduced after fermentation.

According to the odor characteristics of these volatile components, it could be roughly classified into seven categories, namely, herbal, green, floral, fruity, sweetish, toasty, and other. As shown in Figure 3, type 1 contained a large number of substances that presented floral and fruity odors as well as a few substances that presented sweetish and toasty flavors. The overall odor was rich, especially fruity and floral scents. Type 2 covered all categories sharing comparable proportions except fruit and sweetish odors. Compared with other substances, floral, fruity, and sweetish chemicals accounted for a much less percentage in type 3, which is attributed to a faint overall odor. Above all, our experiment found that the volatile flavor of green tea infusion could be significantly improved after the fermentation of *P. sajor-caju*.

**Figure 4:**
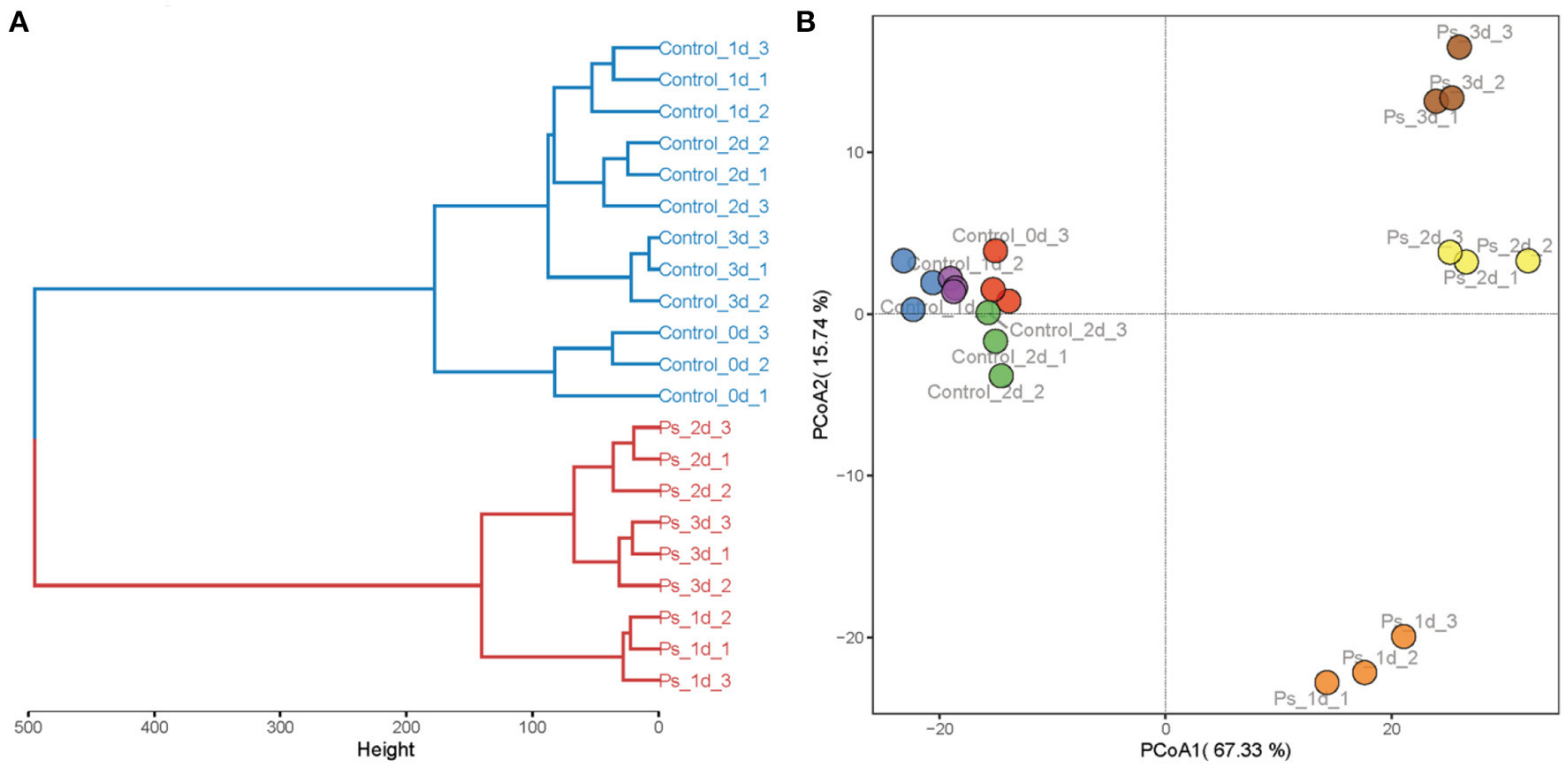
Hierarchical clustering analysis **(A)** and principal component analysis **(B)** of the volatile compounds in tea infusion (Control: unfermented green tea infusion; Ps: green tea infusion fermented by *Pleurotus sajor-caju*; time: from 1 to 3 days).

Based on the concentrations of all volatile components in green tea infusion samples, HCA and PCA were further performed to clarify diversities among them, as shown in Figure 4. In HCA, the volatile components were clustered into two main groups, namely, tea infusion before fermentation and after fermentation. The hierarchical clustering dendrogram is shown in Figure 4A. The PCA score plot was further performed for the dynamic changes of volatile components during the fermentation process (Figure 4B). The variances of the first two major components were 67.05% and 26.31%, which collectively account for 93.36% of the total variability of volatile compounds. The data made the information of the volatile components in each tea sample clear. The PCA scatter plot demonstrated that tea infusion before fermentation had a similar volatile profile. Combining the results of their same cluster identified in HCA, it can be concluded that during the fermentation time the flavor of unfermented tea infusion slightly changed within 3 days. Remarkably, the fermentation processes had changed the profiles of tea infusion volatile greatly, which lead to a distinctive scatter of the sample. The volatile profile was mainly composed of (*Z*)-3-hexen-1-ol and linalool oxide after 1-day fermentation, whereas it turned to be linalool, geraniol, and methyl salicylate and other substances in a few days.

### Significant Changes in Volatile Flavor Components After Fermentation

Significant analysis was performed to further clarify whether the changes of volatile components of the tea soup after fermentation were significant. A total of 54 volatile components of the experimental group showed significant difference compared with the control group after fermentation of 3 days. Among them, in the experimental group, 30 substances such as geraniol, linalool, (*R*)-citronellol, β-damascenone, methyl salicylate, and dihydroactinidiolide were increased remarkably (*p* < 0.01). They mainly had a floral aroma (e.g., linalool, geraniol and (*R*)-citronellol), fruity aroma (e.g., dihydroactinidiolide), and sweetish flavor (e.g., methyl salicylate). The higher the content, the richer the fragrance. In the control group, 24 components, including indole, hexanoic acid, nonanoic acid, hexadecane, and theaspirane, were at a significantly higher level. Table 1 lists several vital volatiles in tea infusion before and after fermentation by *P. sajor-caju*.

**Table 1:** Volatile flavor components that changed significantly after fermentation by *Pleurotussajor*−*caju*^a^.

**No**.	**Compounds**	**Odor description**	**RI**	**Content (μg/L)**	
				**Control-3d**	**Ps-3d**
1	Geraniol	Rose-like, citrus-like	1805	1.20 ± 0.11	12.38 ± 0.96^**^
2	Linalool	Floral, fruity	1535	0.75 ± 0.08	9.19 ± 0.57^**^
3	Benzeneacetaldehyde	Floral, honey, sweet, chocolate-like	1649	0.19 ± 0.01	0.92 ± 0.02^**^
4	Benzaldehyde	Fruity, nutty, woody	1518	0.30 ± 0.02	1.18 ± 0.13^**^
5	β-damascenone	Sweet, honey, apple-like, rose-like	1789	0.10 ± 0.01	0.43 ± 0.05^**^
6	(*Z*)-3-hexenol	Floral	1399	0.31 ± 0.02	1.75 ± 0.31^**^
7	(*R*)-Citronellol	Floral, sweet, rose, fruity citrus nuances	1774	n.d.	0.39 ± 0.04
8	Methyl salicylate	Fresh, faint gingery, grass and milky	1790	n.d.	2.72 ± 0.02
9	2,5-Dimethylpyrazine	Nutty, coffee, cocoa-like	1350	n.d.	0.49 ± 0.32
10	Methyl benzoate	Herb, lettuce, prune, violet	1638	n.d.	0.24 ± 0.01
11	Nerol	Lemon-like, floral	1785	n.d.	0.20 ± 0.04
12	Dihydroactinidiolide	Roasted, musk, coumarin	2163	n.d.	1.12 ± 0.04
13	β-Ionone	Woody, violet-like	1983	0.20 ± 0.04	n.d.
14	(*Z*)-2-penten-1-ol	Green	1352	0.61 ± 0.02	n.d.
15	methyl heptadienone	Green, slightly herbal	1581	0.23 ± 0.02	n.d.
16	Indole	Floral, animal-like	2132	1.12 ± 0.07	0.22 ± 0.13^**^
17	Nonanoic acid	Fatty, waxy-like, cheesy-like	2140	2.84 ± 0.09	1.00 ± 0.18^**^

*(A) Since the difference in volatile components is the most significant when fermenting for 3 days, the content in the figure is presented for 3 days. (B) Odor descriptions were from the literature (38–41), or from FEMA database. (C)*

a *Data are expressed as mean ± SD (n = 3)*,

** *p value of before vs. after fermentation was <0.01*.

### Changes of Antioxidant Activity in Tea Infusion Before and After Fermentation

**Figure 5:**
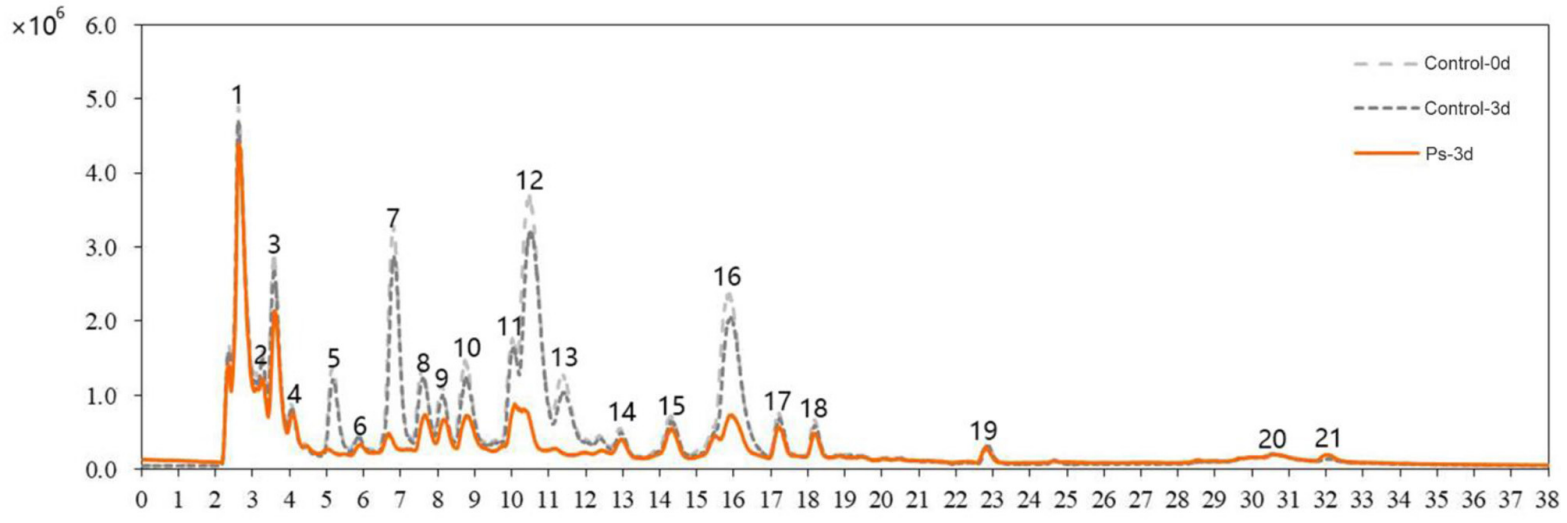
TIC chromatogram of green tea infusion before and after fermentation.

**Figure 6:**
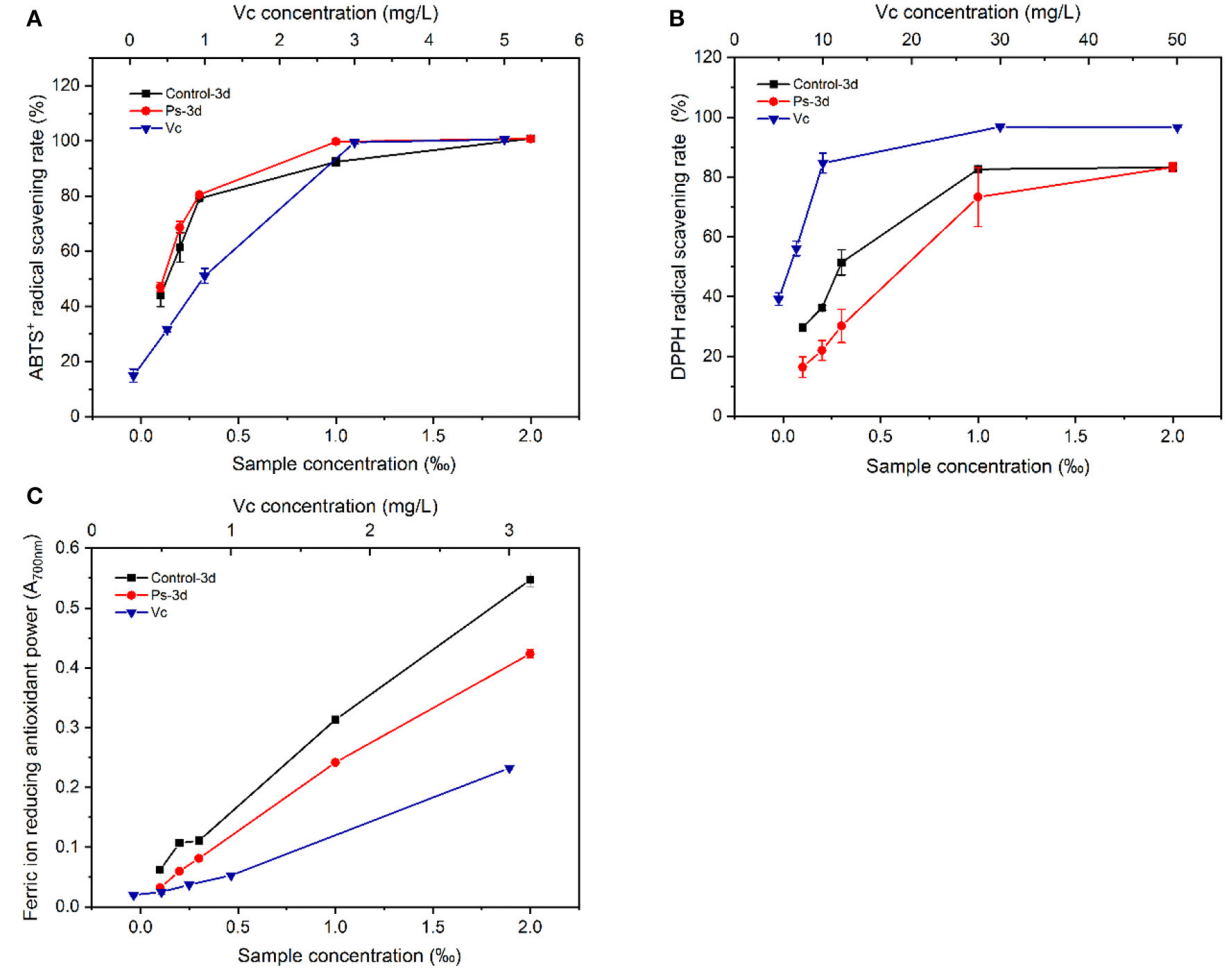
Comparison of antioxidant activity between unfermented green tea infusion and the tea infusion fermented by *Pleurotus sajor-caju* [**(A)** ABTS; **(B)** DPPH; **(C)** FRAP; ferric ion-reducing antioxidant power].

The representative chromatograms before and after tea fermentation obtained from the LC-MS are demonstrated in Figure 5. A total of 21 peaks were measured. Compared with the control group, a total of 9 peaks decreased significantly after fermentation based on Mass and MassBank. We identified 15 compounds (Table 2), including 4 catechins (compounds 10, 11, 12, and 16), 1 anthocyanins (compound 7), 5 flavonols (compounds 5, 14, 15, 17, and 18), and 5 phenolic acids (compounds 1, 3, 4, 6, and 8). After fermentation, catechins and anthocyanins were noticeably reduced. In contrast, any differences cannot be seen in the level of flavonols (except for compound 5) and phenolic acids. ABTS-free radical-scavenging rate, DPPH-free radical-scavenging rate, and ferric iron-reducing antioxidant power were used as indicators to measure the change of antioxidant activity before and after fermentation (Figure 6). Compared with the control group, the ABTS-free radical-scavenging activity in tea infusion fermented for 3 days was slightly increased (Figure 6A) and the calculated Vc equivalents were 11.26 mg/ml (IC_50_ = 0.05‰) and 14.65 mg/ml (IC_50_ = 0.08‰), respectively; the DPPH-free radical-scavenging activity after fermentation was significantly decreased (Figure 6B), and the calculated Vc equivalents were 20.49 mg/ml (IC_50_ = 0.34‰) and 12.08 mg/ml (IC_50_ = 0.50‰), respectively; the ferric ion-reducing power of the experimental group showed a downward trend (Figure 6C). Although the antioxidant activity was lost, it still maintained a high activity after the fermentation of *P. sajor-caju*.

**Table 2:** LC-MS qualitative analysis of polyphenols and anthocyanins in green tea infusion before and after the fermentation by *P. sajor-caju*.

**Peak No**.	**Tentative identification**	**Measured** **[M-H]^−^(m/z)**
1	Quinic acid	191.05
3	Theogallin	343.07
4	Gallic acid	169.01
5	Cyanidin-3,5-di-O-glucoside	611.16
6	Caffeoylquinic acid	353.08
7	2,3-(*E*)-3,4-(*E*)-Anthocyanin	306.07
8	*p*-Coumaroylquinic acid	337.10
10	Procyanidin B1	557.14
11	Epicatechin	289.07
12	Epigallocatechin gallate	458.08
14	Quercetin-hexosyl-hexosyl-deoxyhexoside	771.20
15	Rutin	609.15
16	Epicatechin gallate	442.09
17	Kaempferol-hexosyl-hexoside	593.15
18	Kaempferol-hexoside	447.09

## Discussion

The aroma profile of green tea infusion showed remarkable differences before and after fermentation. Herbal and green, as two original aromas of green tea, are stronger in the unfermented tea infusion. After fermentation, these two odors became much weaker mainly due to the reduction in green-like substances such as (Z)-2-penten-1-ol and methyl heptadienone. In fermented tea infusion, the floral and fruity aroma are enhanced significantly, especially the floral odor. From the results of Figures 2, 4, it can be found that after fermentation of *P. sajor-caju*, linalool (orange, lemon, floral), geraniol (floral, rosy), β-damascenone (apple, rose), (R)-citronellol (floral, rose, citrus), nerol (sweet natural neroli citrus magnolia), citral (lemon sweet), benzaldehyde (sweet, cherry, almond), and phenethyl alcohol (floral, sweet, rosey) increased significantly. The above aromas are predominantly presented floral and fruity. Thereinto, methyl salicylate (sweet taste), displaying a sweetish odor, is a newly added ingredient after fermentation. The toasty odor has a slight variation during fermentation. This kind of odor is mostly derived from the caramel aroma of pyrazine and pyrrole (42), and the composition of such substances has no significant difference before and after fermentation, except for the newly formed 2,5-dimethylpyrazine during fermentation. In addition, analyzing changes in categories of volatile compound after fermentation revealed that aldehydes, esters, and benzene aromatic substances increased, acids and ketones decreased, and there was no significant difference in other types. The l-phenylalanine contained in green tea can be converted into benzene aromatic substances through the action of amino acid decarboxylase and aminotransferase (43). Edible fungi can produce extracellular enzymes such as laccase, lipoxygenase, and hydroperoxide lyase, which may peroxidize and degrade unsaturated fatty acids to form aldehydes (38), and acids will degrade as an immediate carbon source for fungal growth (12).

After fermentation of *P. sajor-caju*, components such as geraniol, linalool, benzaldehyde, β-damascone, and methyl salicylate were significantly increased, imparting floral, fruity, honey-like scent, and have long been recognized as important contributors to the flavor and aroma of various kinds of tea. Among them, geraniol, linalool, and (*Z*)-3-hexen-o-l increased by 9.3, 11.3, and 4.6 times, respectively. Therefore, the strong floral, fruity, and sweetish scents in the after-fermentation infusion were largely improved, which was documented similar to the odor impression as sniffed in fermented green tea infusion (10, 44). Nevertheless, no detailed information regarding the biosynthesis of the odorants in *P. sajor-caju* has been reported so far. Geraniol can be obtained by decomposition of carotenoids; also, geraniol, linalool, and methyl salicylate can also be obtained by enzymatic hydrolysis of the corresponding glycosides. Studies have shown that an increase in linalool, geraniol, (*Z*)-3-hexen-1-ol, and methyl salicylate is related to the effect of β-glucosidase (30). Among the extracellular enzymes produced by *P. sajor-caju*, β-glucosidase activity is high (45–47), and similar results were observed in this study (Supplementary Figure S1). This shows that β-glucosidase can promote the accumulation of the above four substances. Benzaldehyde, as one of the characteristic volatiles of black tea, has an intense nutty scent. Its content increases significantly in the green tea infusion fermenting process, which can be formed by phenylalanine pathway and liberated from glycosidic precursors. Moreover, β-damassterone is a major aroma constituent in green tea, and its content is detected to increase significantly, which may be due to the enzymatic and nonenzymatic degradation of carotenoids, as well as liberation from glycosidic precursors. Further enzymatic cleavage of glycosides and the degradation of carotenoids by *P. sajor-caju* may be the reasons for the increase in β-damassterone concentration during fermentation. Nevertheless, the concentration of β-ionone, which is another kind of carotenoid cleavage product, decreased with fermentation. One reason could be the enzyme system of *P. sajor-caju* preferring the catabolic pathway of carotenoids toward β-damassterone rather than β-ionone. The production of β-damassterone consumes a large amount of β-carotene, which hinders the production of β-ionone (14, 42, 48). Another reason may be no β-ionone glycosides in the tea infusion after it was fermented by *P. sajor-caju*. β-Ionone was oxidized to dihydroactinidiolide by enzymes during fermentation. There are two possible reasons for the formation of this secondary oxidation product, i.e., one is directly generated by secondary enzymatic oxidations from carotenoid precursors, and the other is due to the further oxidation of β-ionone, which may also be the reason for the disappearance of β-ionone after fermentation.

In particular, we also observed that (*R*)-citronellol, methyl salicylate, and 2,5-dimethylpyrazine were newly generated during fermentation. It was reported by Baebara et al. that (*R*)-citronellol could attenuate caffeine bitterness (49). It is worth noting that the (*R*)-citronellol that was newly formed in fermentation may have improved the flavor of tea infusion. 2,5-Dimethylpyrazine is derived from Maillard-type reactions, imparting an intensely roasted, nutty, cocoa-like, chocolate-like scent (35), which was newly formed in fermentation. Marina et al. first highlighted the contribution of vitamin B1 and sodium acetate to the formation of 2,5-dimethylpyrazine during the enokitake fermentation (11). However, further investigations on the formation mechanism of the 2,5-dimethylpyrazine in *P. sajor-caju* are warranted. Methyl salicylate is present only in teas with a fermentation degree of at least semi-fermented, and it cannot be detected in unfermented teas and lightly fermented teas (34), while green tea is unfermented tea without methyl salicylate. It means that methyl salicylate is produced by fermented tea infusion of *P. sajor-caju*. Indole is often accompanied by a spicy pungent odor in green tea, while low-concentration indole presents an aromatic odor. The indole in the experimental group shows a downward trend, which is more conducive to the presentation of a pleasant odor.

To clarify the causes of the abovementioned changes in antioxidant activity (Figure 5 and Table 2), we carried out the LC-MS analysis on the tea infusion in the process of fermentation, and the results showed that a total of 21 peaks were obtained. Compared with the control group, nine peaks were significantly reduced after fermentation. Then, compared with the MassBank database (http://www.massbank.jp/Search) with *m*/*z*, it was found that catechins, including EC, EGCG, ECG, and procyanidin B1, were significantly reduced in the fermented tea infusion. Tea polyphenols with catechins as the key contributor of antioxidant activity are astringent, bitter, and water-soluble compounds. These substances not only reduce the antioxidant capacity of fermented tea but also lead to the decrease in bitterness and astringency of tea (10, 23). This is mainly due to the oxidation of polyphenol oxidase and peroxidase produced in the fermentation of *P. sajor-caju*. After the action of these two enzymes, catechin will be converted into catechin monoquinone, and these quinones or monomers of quinones and catechin will be coupled and polymerized to form catechin polymers, such as theaflavins, thearubins, and tan (50). In this study, it was observed that the activities of these two enzymes gradually increased during the fermentation (*p* < 0.01) (Supplementary Figure S2), and the color of the tea infusion deepened (Supplementary Figure S3), which also confirmed the occurrence of the oxidation process. Furthermore, we found that EGCG containing a pyrogallol-type B-ring declined the most during the fermentation, possibly due to the fact that fungal enzymes might prefer to degrade the pyrogallol-type B ring for degradation, which is similar to the study by Zhang et al. (10).

At present, many previous works indicated that anthocyanins exhibit a good antioxidant activity (51, 52). It is a glycoside derivative, and its reduction may be related to the role of glycosidase, which further reduces the resistance of antioxidant activity. Furthermore, glycoside precursors were found from several components identified, and their reduction is inseparable from the role of glycosidases. *P. sajor-caju* can produce glycosidases to decompose glycosides to release aromatic substances and enhance the sense quality of green tea. Compared with the control group, the ABTS-free radical-scavenging ability was improved to a certain extent after fermentation with the *P. sajor-caju*, and the DPPH-free radical-scavenging power as well as the ferric-reducing antioxidant power had less loss of antioxidant activity. From the results of LC-MS, it can be found that catechins and anthocyanins with antioxidant activity declined significantly, whereas the contents of theaflavins, thearubins (53–55), and volatile components such as linalool and geraniol (56–58), which also have an antioxidant activity, were increased. Based on the findings of this study, these substances may affect the antioxidant activity and need to be further investigated.

## Conclusion

Green tea infusion usually has distinct astringency, bitter taste, and typical green flavor as a consequence of its post-harvest treatment without withering and enzymatic oxidation. In this study, the innovative approach driven by edible mushroom *P. sajor-caju* has achieved promising potential to manipulate the green tea flavor during the fermentation process. Herbal and grass odorants produced by green-like (*Z*)-2-penten-1-ol and methyl heptadienone were decreased significantly as a result of the fermentation with *P. sajor-caju* mycelium (*p* < 0.01). In contrast, the contents of linalool and geraniol infusion were increased 9.3 and 11.3 times, respectively, whereas methyl salicylate newly produced during this process augmented pleasant flower and fruit flavors. Moreover, polyphenol profile performed by HLPC-MS revealed that the contents of most polyphenols in green tea infusion declined markedly. Due to the noticeable reduction of catechins and anthocyanins in fermented green tea infusion, the astringency and bitterness were significantly improved. There was an obvious decrease in antioxidant activity, especially the DPPH-free radical-scavenging ability and the ferric-reducing power. It is noteworthy that the ABTS-free radical-scavenging ability of green tea infusion was improved by *P. sajor-caju* fermentation to a certain extent, indicating that the increased tea pigments and volatile metabolites after fermentation with *P. sajor-caju* may contribute to the antioxidant capacity of green tea infusion. Based on the findings of this study, further studies on the *in vivo* antioxidant activity of flavor compounds in animal experiments are required.

## Data Availability Statement

The original contributions presented in the study are included in the article/Supplementary Material, further inquiries can be directed to the corresponding author/s.

## Author Contributions

W-YS: conceptualization, methodology, investigation, and writing—original draft. S-YG: validation, investigation, formal analysis, and writing—original draft. S-JZ: writing—original draft and visualization. QW: methodology and validation. G-MC, J-ZH, X-CL, and P-FR : validation, writing—review and editing and supervision. LN: methodology, validation, writing—review and editing, supervision, and project administration. All authors contributed to the article and approved the submitted version.

## Funding

This study was financially supported by the National Natural Science Foundation of China, Science Foundation of Two Sides of Strait (No. U2005209).

## Conflict of Interest

The authors declare that the research was conducted in the absence of any commercial or financial relationships that could be construed as a potential conflict of interest.

## Publisher's Note

All claims expressed in this article are solely those of the authors and do not necessarily represent those of their affiliated organizations, or those of the publisher, the editors and the reviewers. Any product that may be evaluated in this article, or claim that may be made by its manufacturer, is not guaranteed or endorsed by the publisher.
