# Supplementary material for: Evaluation of Volatile Profile and In Vitro Antioxidant Activity of Fermented Green Tea Infusion With Pleurotus sajor-caju (Oyster Mushroom)
Source: Front Nutr. 2022 Apr 14;9:865991. doi: 10.3389/fnut.2022.865991 (PMC9047879; doi:10.3389/fnut.2022.865991)
Supplement: Supplementary file 1 [file Data_Sheet_1.docx]

Supplementary Materials

# List of Supplementary Figures


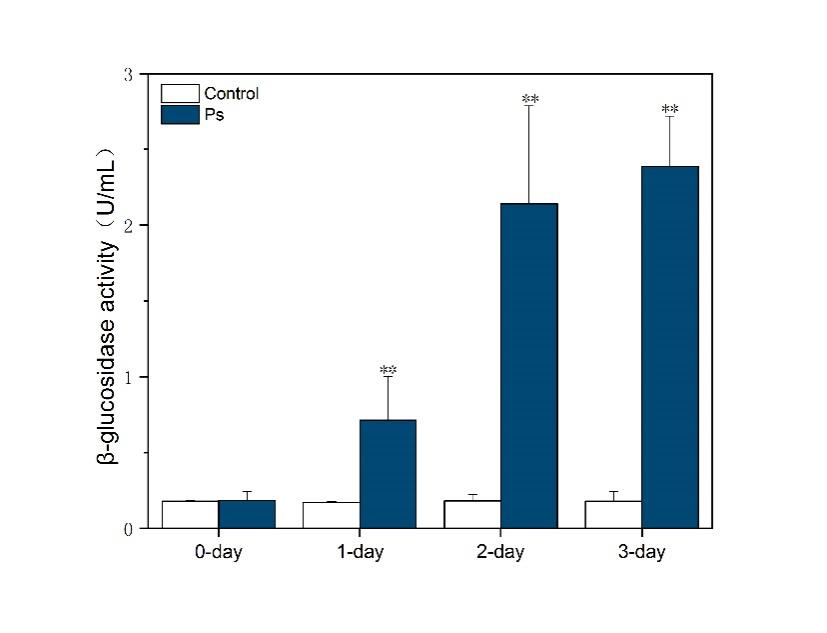


**Fig. S1.** Determination of *β*-glucosidase activity in green tea infusion before and after fermentation (n=3). ** Indicates significant difference in *t*-test (*p* < 0.01)


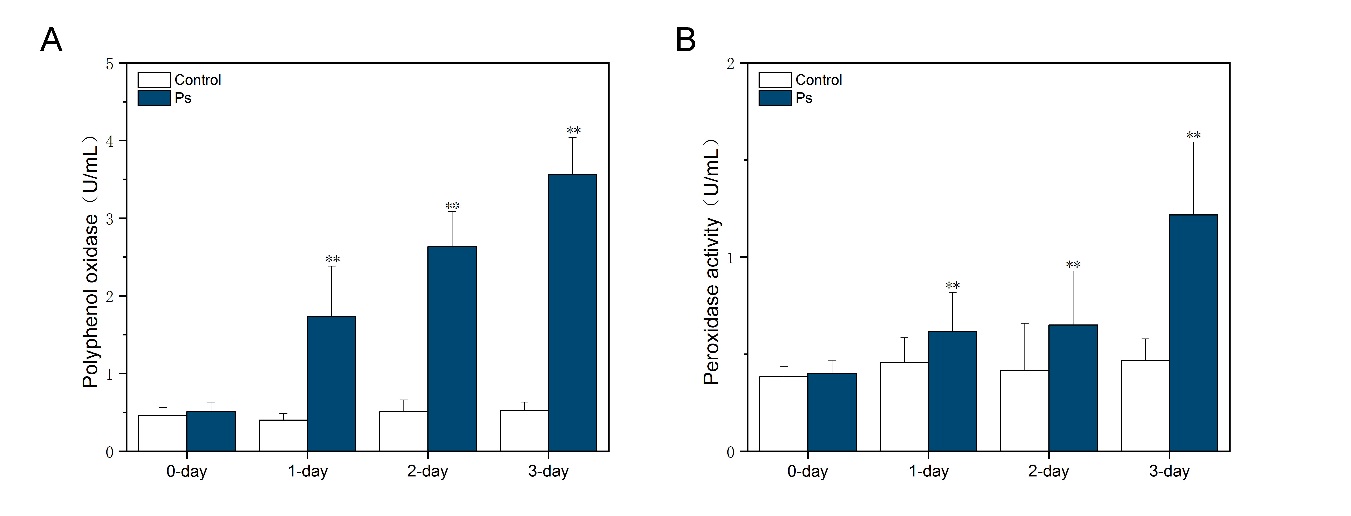


**Fig. S2.** Determination of polyphenol oxidase(A) and peroxidase(B) activity in green tea infusion before and after fermentation by *Pleurotus sajor-caju* (n=3). ** Indicates significant difference in *t*-test (*p* < 0.01)

**
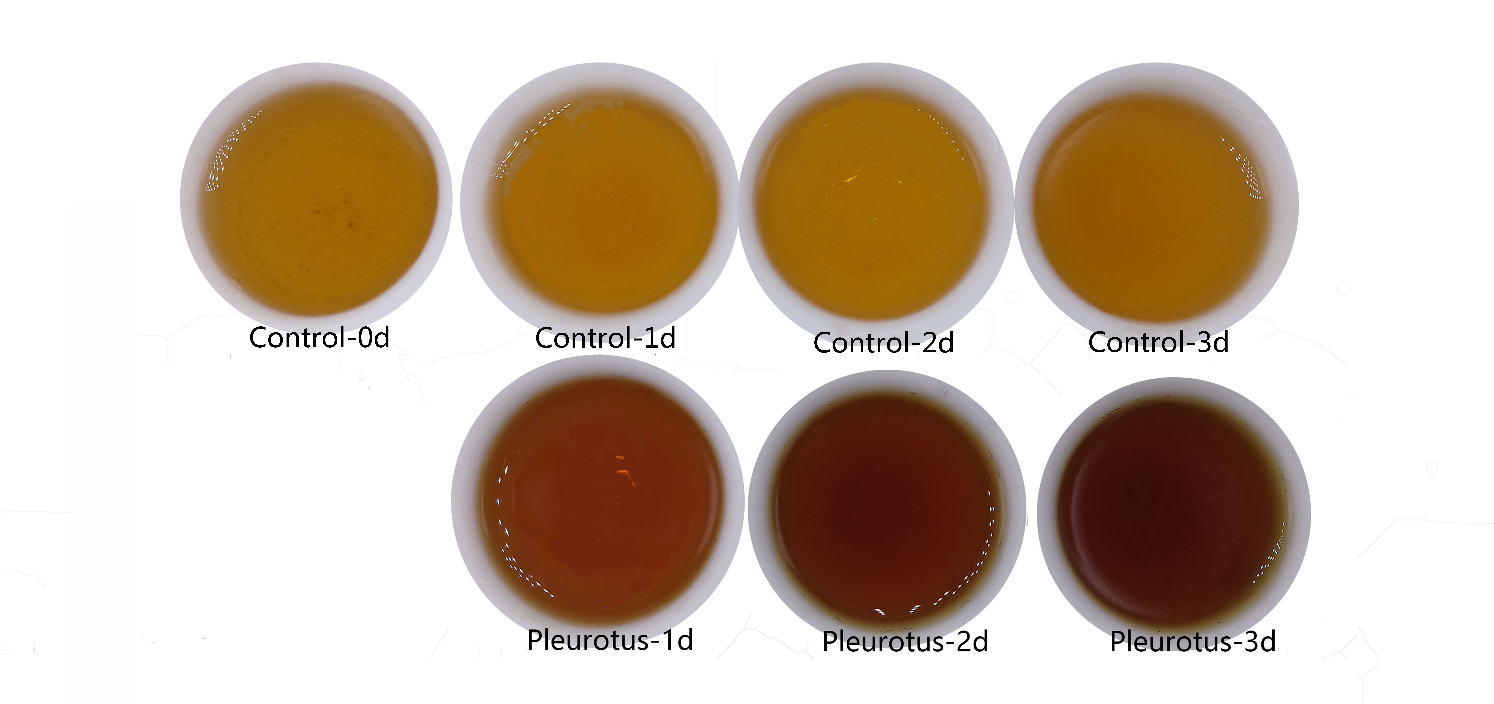
**

**Fig. S3.**  Changes of tea infusion’s color before and after fermentation by *Pleurotus sajor-caju*.
